# Simultaneous degradation of aflatoxin B_1_, zearalenone, and deoxynivalenol by *Trametes versicolor* laccases

**DOI:** 10.3389/fnut.2026.1834914

**Published:** 2026-06-03

**Authors:** Pengna Li, Yilan Wang, Yang Li, Reham Ahmed Khashaba, Qiongqiong Li, Haiwei Lou, Renyong Zhao

**Affiliations:** 1 College of Food Science and Engineering, Henan University of Technology, Zhengzhou, China; 2 Faculty of Agriculture, New Valley University, Kharga, Egypt

**Keywords:** aflatoxin B_1_, deoxynivalenol, laccase, multi-mycotoxin contamination, *Trametes versicolor*, zearalenone

## Abstract

**Background:**

Multi-mycotoxin co-contamination in staple grains poses a major challenge to food quality and safety, underscoring the urgent need for detoxification approaches capable of simultaneously degrading multiple mycotoxins. This study investigates the potential of *Trametes versicolor* laccase as a biocatalyst for the simultaneous degradation of aflatoxin B_1_ (AFB_1_), zearalenone (ZEN), and deoxynivalenol (DON).

**Methods:**

Single-factor experiments and response surface methodology were used to optimize fermentation conditions for *T. versicolor* strain Tv-1 under submerged fermentation to produce laccase. The secreted laccase was purified using ammonium sulfate fractionation, dialysis, and anion exchange chromatography. Enzymatic properties, including optimal temperature, pH, thermal stability, and pH stability, were analyzed to determine suitable conditions for mycotoxin degradation.

**Results:**

Laccase activity in the fermentation broth reached a maximum of 6843.14 U/L after 7.9 days at 28°C, with an initial medium pH of 5.4 and a medium volume of 105 mL. *T. versicolor* laccase exhibited maximum activity at 50°C and pH 4.5, but for practical detoxification purposes, 30°C and pH 4.5 were selected based on both stability and enzymatic activity considerations. Using laccase preparation S3, simultaneous degradation of AFB_1_, ZEN, and DON was achieved under mediator-free conditions, with degradation rates of 32.88, 91.88, and 54.45%, respectively.

**Conclusion:**

These findings demonstrate that *T. versicolor* laccase can serve as a mediator-free and environmentally friendly biocatalyst for the simultaneous degradation of AFB_1_, ZEN, and DON. However, degradation efficiency decreased at higher initial mycotoxin concentrations and under multi-mycotoxin coexistence conditions, indicating that further optimization is still required for practical applications.

## 1 Introduction

Mycotoxin contamination remains a persistent challenge for global food and feed safety, particularly in staple grains and their derived products. Under real agricultural production and storage conditions, staple commodities such as maize, rice, and wheat are frequently contaminated with two or more mycotoxins rather than a single mycotoxin, resulting in multi-mycotoxin co-contamination and increased toxicological risks (1, 2). Among the major regulated mycotoxins, aflatoxin B_1_ (AFB_1_), zearalenone (ZEN), and deoxynivalenol (DON) are of particular concern due to their high prevalence and severe toxicological profiles (3, 4). AFB_1_ is well known for its strong hepatotoxicity, mutagenicity, and carcinogenicity (5); ZEN exhibits strong estrogenic activity, leading to reproductive disorders, developmental abnormalities, and infertility (6); and DON inhibits protein synthesis and is associated with gastrointestinal toxicity, immune dysregulation, and growth suppression (7). Contamination by these mycotoxins not only leads to substantial economic losses but also poses serious risks to public health. Their frequent co-occurrence in staple commodities further complicates detoxification and highlights the need for effective broad-spectrum mitigation strategies.

Conventional detoxification approaches, including physical separation (8), adsorption (9), and chemical treatment (10), are often constrained by practical drawbacks such as incomplete mycotoxin removal, possible desorption *in vivo*, nutrient losses, and chemical residues (11). In contrast, biological detoxification—especially enzymatic degradation—has gained increasing attention as a promising alternative because of its high specificity, efficiency, mild operating conditions, environmental compatibility, and sustainability (12, 13). To date, multiple enzymes, including manganese peroxidases (14), lactonases (15), esterases (16), and lipases (17), have been investigated for their ability to degrade specific mycotoxins. However, most characterized enzymes exhibit narrow substrate specificity, limiting their utility in real-world scenarios where multiple mycotoxins frequently co-exist. Therefore, there is an urgent need to develop enzymes with a broad substrate spectrum capable of simultaneously degrading multiple major mycotoxins.

Laccase (EC 1.10.3.2), a multicopper oxidase widely produced by white-rot fungi, catalyzes the oxidation of a variety of aromatic compounds, phenolics, and certain non-phenolic substrates, with water as the only by-product, and is therefore often regarded as a “green biocatalyst” (18). Its broad substrate range suggests strong potential for application in multi-mycotoxin detoxification. Previous studies have demonstrated that laccases from different sources can degrade individual mycotoxins; for example, laccases from *Pleurotus eryngii* have been reported to degrade AFB_1_ (19), and *Trametes versicolor* laccase can degrade ZEN (20). However, DON is generally more difficult to degrade. Laccase from *P. eryngii* has been reported to be unable to degrade DON (19), whereas *T. versicolor* laccase achieved DON degradation only in the presence of a chemical mediator such as 2,2,6,6-tetramethylpiperidine-N-oxyl (TEMPO) (21). The use of mediators may increase processing costs and raises concerns regarding potential residual chemicals. Moreover, most enzymatic detoxification studies still focus on single-toxin systems (22), while reports of a single enzyme capable of simultaneously degrading multiple mycotoxins remain limited. In particular, investigations into the simultaneous degradation of AFB_1_, ZEN, and DON by a single mediator-free laccase under mild conditions are scarce.

Therefore, in the present study, a high laccase-producing, edible and medicinal *T. versicolor* strain Tv-1 was employed for laccase production via submerged fermentation. Fermentation conditions for laccase production were optimized through a combination of single-factor experiments and response surface methodology to enhance laccase yield. The laccase was subsequently separated and purified, and the number of laccase isoforms and their enzymatic properties were determined. Finally, we systematically investigated the efficacy of this *T. versicolor* laccase in degrading AFB_1_, ZEN, and DON both individually and simultaneously in a mixed system. This work aims to develop a green, low-temperature, mediator-free, environmentally friendly, and sustainable strategy with strong potential for the simultaneous detoxification of three major mycotoxins.

## 2 Materials and methods

### 2.1 Media and reagents

Potato dextrose agar (PDA) medium was prepared as previously described (23) and used for strain maintenance.

The optimized medium (OM) for laccase production consisted of potato extract (200.00 g/L), maltose (19.30 g/L), yeast extract (2.00 g/L), wheat bran (42.60 g/L), CuSO_4_ (0.48 mmol/L), and KH_2_PO_4_ (3.00 g/L). The medium was sterilized at 121°C for 30 min and then cooled to room temperature prior to use.

Ammonium sulfate (analytical grade) was purchased from Chengdu Aike Reagent Co., Ltd. (Chengdu, China). Methanol and acetonitrile (HPLC grade) were obtained from Shanghai Macklin Biochemical Technology Co., Ltd. (Shanghai, China). Aflatoxin B_1_ (AFB_1_), zearalenone (ZEN), and deoxynivalenol (DON) were purchased as certified reference standards from J&K Scientific Ltd. (Beijing, China).

### 2.2 Strain

The *T. versicolor* strain Tv-1 was purchased from Gaoyou Lianyi Edible Fungi Technology Promotion Center and was used in this study. The strain was maintained on PDA slants at 4°C.

For activation, the strain was inoculated onto fresh PDA plates and incubated at 25°C for 7 days. The activated mycelia were then used as inoculum for subsequent submerged fermentation (SmF) experiments.

### 2.3 Determination of laccase activity

Laccase activity was determined using guaiacol as the substrate according to a previously reported method (23). 2.4 mL of succinic acid buffer (pH 4.5) containing 1.25 mmol/L guaiacol was mixed with 0.6 mL crude laccase solution and incubated at 30°C for 30 min. The absorbance was measured at 465 nm to determine laccase activity. One unit (U) of laccase activity was defined as the amount of enzyme required to oxidize 1 μmol of guaiacol per minute under the assay conditions.

### 2.4 Determination of AFB_1_, ZEN, and DON contents

The contents of AFB_1_, ZEN, and DON were determined by high-performance liquid chromatography (HPLC), following the corresponding Chinese National Standards. Specifically, AFB_1_, ZEN, and DON were quantified according to GB 5009.22–2016 (Determination of aflatoxins B and G in food), GB 5009.209–2016 (Determination of zearalenone in food), and GB 5009.111–2016 (Determination of deoxynivalenol in food), respectively.

The mycotoxin degradation rate was calculated using the following (Equation 1):


Mycotoxindegradationrate(%)=(1-A/B)×100% (1)

where A represents the residual amount of mycotoxin after laccase treatment (μg), and B represents the initial amount of mycotoxin before laccase treatment (μg).

The HPLC analyses were performed using an LC-2040C 3D ultra-high-performance liquid chromatography (UHPLC) system (Shimadzu Corporation, Kyoto, Japan). Chromatographic separation was achieved on a Shim-pack GIST C_18_ column (3.0 mm × 75 mm, 2 μm; Shimadzu Corporation). AFB_1_ and ZEN were detected using a fluorescence detector (RF-20A, Shimadzu Corporation, Kyoto, Japan), while DON was detected using the ultraviolet (UV) detector of the LC-2040C system (Shimadzu Corporation, Kyoto, Japan).

The detailed HPLC conditions were as follows. For AFB_1_ analysis, the mobile phase consisted of water and methanol at a ratio of 55:45 (v/v), and isocratic elution was used. The flow rate was 0.2 mL/min, the injection volume was 10 μL, and the column temperature was maintained at 40°C. AFB_1_ was detected using a fluorescence detector with excitation and emission wavelengths of 365 and 436 nm, respectively. The linearity range was 1–100 ng/mL. According to GB 5009.22–2016, the limit of detection (LOD) and limit of quantification (LOQ) were 0.03 and 0.1 μg/kg, respectively.

For ZEN analysis, the mobile phase consisted of acetonitrile, water, and methanol at a ratio of 46:46:8 (v/v/v), and isocratic elution was used. The flow rate was 0.2 mL/min, the injection volume was 10 μL, and the column temperature was maintained at 30°C. ZEN was detected using a fluorescence detector with excitation and emission wavelengths of 274 and 440 nm, respectively. The linearity range was 10–500 ng/mL. According to GB 5009.209–2016, the LOD and LOQ were 5 and 17 μg/kg, respectively.

For DON analysis, the mobile phase consisted of water and methanol at a ratio of 80:20 (v/v), and isocratic elution was used. The flow rate was 0.2 mL/min, the injection volume was 10 μL, and the column temperature was maintained at 35°C. DON was detected at a UV wavelength of 218 nm. The linearity range was 100–5,000 ng/mL. According to GB 5009.111–2016, the LOD and LOQ were 100 and 200 μg/kg, respectively. Matrix-matched spike-recovery validation was not performed in the present study, which should be addressed in future work involving real food and feed matrices.

### 2.5 Optimization of culture conditions by single-factor experiments

Mycelial plugs (8 mm in diameter) were cut from activated colonies of *T. versicolor* strain Tv-1 grown on PDA plates. Five plugs were inoculated into 250 mL Erlenmeyer flasks containing the optimized medium (OM) and incubated in a constant-temperature shaking incubator (Shanghai Zhichu Instrument Co., Ltd., Shanghai, China) under dark conditions. The effects of fermentation time, temperature, initial pH of the medium, and medium volume on laccase production were investigated.

#### 2.5.1 Effect of fermentation time on laccase production

To evaluate the effect of fermentation time, cultures were incubated at 25°C with an initial medium pH of 5.5, a medium volume of 100 mL, and an inoculum size of five mycelial plugs (8 mm in diameter). Culture broth samples were collected at various fermentation time points (2, 4, 6, 8, 10, 12, 14, and 16 days), centrifuged, and the supernatants were used to determine laccase activity.

#### 2.5.2 Effect of fermentation temperature on laccase production

The effect of fermentation temperature on laccase production was examined by incubating the cultures at different temperatures (22, 25, 28, 31, 34, and 37°C). The fermentation time was fixed at 8 days. The initial medium pH, medium volume, and inoculum size were set to 5.5, 100 mL, and five mycelial plugs (8 mm in diameter), respectively.

#### 2.5.3 Effect of initial medium pH on laccase production

To investigate the effect of initial medium pH on laccase production, cultures were incubated at 25°C for 8 days with a medium volume of 100 mL and an inoculum size of five mycelial plugs (8 mm in diameter). The medium pH was adjusted to 2.5, 3.5, 4.5, 5.5, 6.5, 7.5, 8.5, 9.5, and 10.5 prior to sterilization.

#### 2.5.4 Effect of medium volume on laccase production

The effect of medium volume on laccase production was evaluated by incubating cultures with different medium volumes (40, 70, 100, 130, and 160 mL) in 250 mL Erlenmeyer flasks. The fermentation time and temperature were fixed at 8 days and 25°C, respectively, while the initial medium pH and inoculum size were set at 5.5 and five mycelial plugs (8 mm in diameter) per flask, respectively.

### 2.6 Optimization of culture conditions by response surface methodology

Based on the single-factor experiments, fermentation time (*X*_1_), initial medium pH (*X*_2_), and medium volume (*X*_3_) were selected as the key process variables for response surface optimization. Laccase activity in the fermentation broth was measured as the response (*Y*). A Box–Behnken design was used to optimize the fermentation conditions for laccase production by *T. versicolor* strain Tv-1. During the optimization experiments, the inoculum size was fixed at five mycelial plugs (8 mm in diameter), and the fermentation temperature was maintained at 28°C. The coded levels of the three variables were determined based on the single-factor results and are presented in Table 1.

**TABLE 1:** Factors and levels in the Box–Behnken design for laccase production.

Codes	Factors	Levels		
		−1	0	1
*X*_1_	Fermentation time (day)	6	8	10
*X*_2_	Initial medium pH	4.5	5.5	6.5
*X*_3_	Medium volume (mL)	70	100	130

A Box–Behnken design was performed using Design-Expert software, and the design matrix is presented in Table 2. After conducting the Box–Behnken experiments and performing analysis of variance (ANOVA), the optimal culture conditions for laccase production by *T. versicolor* strain Tv-1 were determined, with the following parameter settings: fermentation time ranging from 6 to 10 days, initial medium pH ranging from 4.5 to 6.5, and medium volume ranging from 70 to 130 mL. The goal value for laccase activity was set to maximum.

**TABLE 2:** Box–Behnken design matrix and results for laccase production.

Run	*X*_1_ Fermentation time (day)	*X*_2_ Initial medium pH	*X*_3_ Medium volume (mL)	*Y* Laccase activity (U/L)
1	−1	−1	0	5028.18
2	1	−1	0	4708.71
3	−1	1	0	4509.62
4	1	1	0	4305.90
5	−1	0	−1	1544.12
6	1	0	−1	1349.65
7	−1	0	1	3782.71
8	1	0	1	3203.96
9	0	−1	−1	2176.10
10	0	1	−1	1865.89
11	0	−1	1	3657.70
12	0	1	1	3514.17
13	0	0	0	6694.98
14	0	0	0	6898.70
15	0	0	0	6676.46
16	0	0	0	6546.82
17	0	0	0	6870.92

### 2.7 Purification of *T. versicolor* laccase

#### 2.7.1 Preparation of crude laccase preparation

*T. versicolor* strain Tv-1 was inoculated into the optimized OM medium and cultured under the optimal conditions established in this study for laccase production. The fermentation broth was harvested and centrifuged at 8,000 r/min for 5 min at 4°C to collect the supernatant containing laccase. The supernatant was then filtered through a 0.45 μm membrane filter to obtain the filtrate, which was designated as the crude laccase preparation (sample S1).

#### 2.7.2 Fractional precipitation of laccase by ammonium sulfate

A 100 mL portion of sample S1 was transferred to a beaker, and ammonium sulfate was gradually added with continuous stirring to achieve saturation levels of 20, 30, 40, 50, 60, 70, 80, and 90%. The mixture was then centrifuged at 4°C and 10,000 r/min for 30 min to separate the supernatant and precipitate. Laccase activity in the supernatant was measured to monitor precipitation of laccase. As ammonium sulfate saturation increased, the laccase activity in the supernatant decreased sharply beyond a certain level, which was defined as the first-stage salting-out level; this step removed a portion of protein impurities. The supernatant from the first-stage salting-out was further fractionated by increasing ammonium sulfate saturation. When the ammonium sulfate saturation exceeded a certain level, laccase activity in the supernatant became very low and no longer decreased markedly with further increases in ammonium sulfate saturation; this point was defined as the second-stage salting-out level. The crude laccase precipitate obtained from the second-stage salting-out was collected by centrifugation and redissolved in 10 mL of phosphate buffer (PB), prepared by mixing 5.3 mL of 0.2 mol/L sodium dihydrogen phosphate (NaH_2_PO_4_) with 94.7 mL of 0.2 mol/L disodium hydrogen phosphate (Na_2_HPO_4_). The resulting solution was designated as the ammonium sulfate–fractionated laccase preparation (S2).

#### 2.7.3 Desalting by dialysis

Laccase preparation S2 was transferred into dialysis tubing with a molecular weight cut-off (MWCO) of 8,000–14,000 Da and dialyzed against PB at 4°C for 24 h. The dialysis buffer was replaced every 3 h to ensure effective removal of residual ammonium sulfate. After dialysis, the dialysis bag was placed in polyethylene glycol (PEG 20000) to concentrate the sample by partial removal of water. The concentrated dialyzed sample was then filtered through a 0.45 μm membrane filter and adjusted to a final volume of 10 mL with PB. The resulting desalted laccase preparation was designated as laccase preparation S3.

#### 2.7.4 Anion-exchange purification

An aliquot (1 mL) of laccase preparation S3 was applied to a DEAE-Sepharose 6 FF anion-exchange column (Beijing Solarbio Science & Technology Co., Ltd., Beijing, China) pre-equilibrated with PB. The column was allowed to stand for 2 min to facilitate protein–resin interaction. Elution was first performed with PB, and the eluate was collected. Proteins remaining on the column were then eluted with PB containing NaCl at stepwise increasing concentrations (0.1–0.6 mol/L). Fractions were collected in 2 mL aliquots.

Laccase activity was measured for each fraction, and protein elution was monitored by measuring absorbance at 280 nm (OD_280_) (24). In the elution profile, the sample corresponding to the first peak showing laccase activity was designated as LacA, and the sample corresponding to the second peak showing laccase activity was designated as LacB. Fractions exhibiting laccase activity were retained for subsequent analysis.

#### 2.7.5 SDS-PAGE and native-PAGE analysis

Sodium dodecyl sulfate–polyacrylamide gel electrophoresis (SDS-PAGE) was performed on 12% polyacrylamide gels to analyze the laccase preparations (S1, S2, S3, LacA, and LacB). The gels were stained with Coomassie Brilliant Blue R-250. Molecular weight determination of LacA and LacB was carried out using a protein ladder (Beijing Solarbio Science & Technology Co., Ltd., Beijing, China) with a molecular weight range of 11–180 kDa, according to the method described previously (25).

For native polyacrylamide gel electrophoresis (Native-PAGE), 12% native polyacrylamide gels were used for zymogram analysis, using a 1.0 mmol/L guaiacol solution for staining (26).

#### 2.7.6 Determination of protein content

Protein concentrations in preparations S1, S2, S3, LacA, and LacB were determined using the Bradford assay (27), with bovine serum albumin (BSA) as the standard protein.

### 2.8 Enzymatic characterization of *T. versicolor* laccase

#### 2.8.1 Optimum temperature and thermal stability

Laccase activity was measured using guaiacol as the substrate, as previously described (23). The reaction mixture had a total volume of 3 mL, with a pH of 4.5 and a reaction time of 30 min. The optimum temperature for laccase activity was determined by incubating the enzyme at temperatures ranging from 20 to 80°C, with the highest activity set as 100%.

To assess thermal stability, *T. versicolor* laccase was incubated at 30, 40, 50, and 60°C for 0, 2, 4, 6, 8, 10, and 12 h. After incubation, laccase activity was measured, and the activity at 0 h was set as 100% to evaluate enzyme stability at different temperatures.

#### 2.8.2 Optimum pH and pH stability

Laccase activity was measured using guaiacol as the substrate, with a reaction mixture of 3 mL, a reaction temperature of 30°C, and a reaction time of 30 min. The optimum pH for laccase activity was determined by incubating the enzyme at pH values ranging from 3.0 to 7.0, with the highest activity set as 100%.

To assess pH stability, *T. versicolor* laccase was incubated in succinic acid–sodium hydroxide buffer at pH values of 3.0, 3.5, 4.0, 4.5, 5.0, 5.5, 6.0, 6.5, and 7.0 for 24 h. Laccase activity was then measured, and the highest activity was set as 100% to evaluate enzyme stability at different pH levels.

### 2.9 Single- and mixed-mycotoxin degradation by *T. versicolor* laccase

Among the laccase preparations obtained in this study, S3 (as described in section 2.7.3) exhibited higher specific activity and a relatively high recovery yield compared to S1 and S2, making it more suitable for mycotoxin degradation. In contrast, the purified isoforms LacA and LacB showed lower recovery yields and were difficult to obtain in sufficient quantities. Therefore, S3 was selected for the mycotoxin degradation experiments.

Based on the thermal stability and optimum pH of *T. versicolor* laccase, degradation of mycotoxins (AFB_1_, ZEN, and DON) was studied at 30°C and pH 4.5. The pH of the reaction mixture was adjusted to 4.5. Using the previously described laccase inactivation method (28), S3 was heat-inactivated by autoclaving at 121°C for 30 min (with no detectable laccase activity). Heat-inactivated laccase preparation S3 was then used as a control in all mycotoxin degradation experiments to determine whether the degradation was enzymatically mediated.

#### 2.9.1 Degradation of individual mycotoxins

AFB_1_, ZEN, and DON stock solutions were prepared by dissolving the standards in methanol (AFB_1_) or acetonitrile (ZEN, DON) to final concentrations of 4 μg/mL (AFB_1_, ZEN) and 100 μg/mL (DON). In each 1 mL reaction mixture, 100 μL of either laccase preparation S3 (44448 U/L, as described in section 2.7.3) or heat-inactivated laccase preparation S3, along with the respective mycotoxin stock solution, were added to achieve final concentrations of 50, 100, and 200 ng/mL for AFB_1_, 300, 600, and 900 ng/mL for ZEN, and 2,000, 3,000, and 4,000 ng/mL for DON, respectively. Reactions were carried out at 30°C for 24, 48, and 72 h.

For AFB_1_ and ZEN degradation, the mycotoxins were extracted from the reaction mixture using dichloromethane (three times, 1 mL each). The solvent was evaporated by nitrogen blow-off, and the residues were redissolved in 1 mL of the mobile phase used for mycotoxin detection (Section 2.4). Solutions were filtered through a 0.22 μm membrane filter, and the filtrate was used for quantification.

For DON degradation, extraction was performed with ethyl acetate (three times, 1 mL each). After solvent evaporation by nitrogen blow-off, the residues were redissolved in 1 mL of the mobile phase used for DON detection (section 2.4), filtered through a 0.22 μm membrane filter, and the filtrate was used for quantification.

#### 2.9.2 Simultaneous degradation of three mycotoxins

A 12.5 μL aliquot of AFB_1_ stock solution (4 μg/mL), 75 μL of ZEN stock solution (4 μg/mL), 20 μL of DON stock solution (100 μg/mL), and 100 μL of either the laccase preparation S3 (44448 U/L) or the heat-inactivated laccase preparation S3 were added to a centrifuge tube. After adding succinic acid–sodium hydroxide buffer, the pH was adjusted to 4.5, and the final volume was brought to 1 mL. The final concentrations of AFB_1_, ZEN, and DON were 50, 300, and 2,000 ng/mL, respectively. The mixture was thoroughly mixed and incubated at 30°C for 24, 48, 72, 96, 120, 144, 168, 192, and 216 h.

For simultaneous degradation, AFB_1_, ZEN, and DON were extracted from the reaction mixture using the methods described in section 2.9.1. After nitrogen blow-off, the residues were redissolved in the corresponding mobile phase for mycotoxin detection (section 2.4), filtered through a 0.22 μm membrane filter, and the filtrate was used for quantification.

All degradation experiments with individual and combined mycotoxins were performed in three independent biological replicates, and the data are presented as the mean ± standard deviation (SD).

### 2.10 Statistical analysis

Line charts were generated using OriginPro 2025 software (OriginLab Corporation, Northampton, United States). Response surface plots were constructed using Design-Expert 8.0.6 software (Stat-Ease Inc., Minnesota, United States) to assess the effects of independent variables on laccase production. Statistical significance was determined using SPSS 27.0 software (SPSS Inc., Chicago, United States), with differences analyzed using Duncan’s multiple range test. The significance level was set at *p* < 0.05.

## 3 Results and discussion

### 3.1 Effects of culture conditions on laccase production by *T. versicolor*

The effects of fermentation time, temperature, initial medium pH, and medium volume on laccase production by *T. versicolor* strain Tv-1 were investigated using single-factor experiments.

#### 3.1.1 Effect of fermentation time on laccase production

**FIGURE 1:**
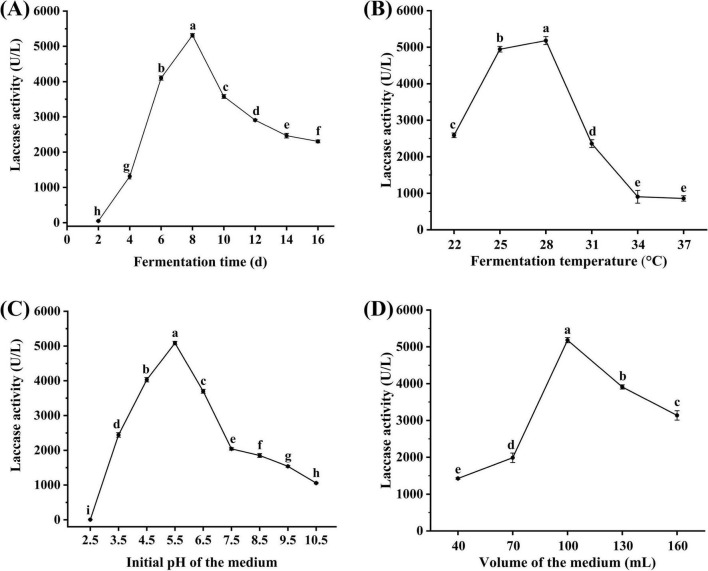
Effects of fermentation time **(A)**, fermentation temperature **(B)**, initial pH of the medium **(C)**, and volume of the medium **(D)** on laccase production by *T. versicolor* strain Tv-1. Different letters indicate significant differences (*p* < 0.05, Duncan’s multiple range test).

The effect of fermentation time on laccase production is shown in Figure 1A. Laccase activity increased markedly during the early stages, reached a peak on day 8, and then declined. From day 2 to day 8, laccase activity rose sharply, likely because continued fungal growth enhanced laccase secretion. This growth-associated pattern is consistent with reports on wood-decaying fungi under submerged fermentation (29). After day 8, laccase activity gradually decreased, possibly due to protease-mediated degradation of the enzyme, nutrient depletion, or the accumulation of inhibitory metabolites (29). Such phenomena are commonly observed during prolonged fungal fermentation (29–31). The optimal fermentation time for maximum laccase activity varies among microorganisms (32). For white-rot fungi, peak laccase production typically occurs between 6 and 20 days, depending on the strain and cultivation conditions (33). For example, *T. versicolor* strain K1 achieved peak activity on day 7 (34), while peak laccase activity was observed on day 9 in scaled-up submerged fermentation (35). In this study, peak activity occurred on day 8, which is broadly consistent with these findings. These results suggest that optimal fermentation time is strain-dependent and influenced by medium composition and culture conditions (32, 35). Therefore, optimizing fermentation time for each strain is essential for maximizing laccase production.

#### 3.1.2 Effect of fermentation temperature on laccase production

The effect of fermentation temperature on laccase production is shown in Figure 1B. Laccase activity increased as the temperature rose from 22 to 28°C and decreased at higher temperatures, with the highest activity observed at 28°C. Microbial growth and metabolism are strongly influenced by temperature, with each strain exhibiting an optimal range. Deviation from this optimal range can reduce metabolic activity and enzyme production, while optimal temperatures promote rapid growth and enhanced biosynthesis (36, 37). For *T. versicolor* strain Tv-1, 28°C appears to be optimal for both growth and laccase secretion. Temperatures above or below this value result in reduced laccase production, likely due to decreased metabolic activity (36). Notably, this optimal temperature is comparable to that reported for *T. versicolor* strain UC-3, which exhibited maximal laccase production at 30°C (33). These results highlight fermentation temperature as a key factor regulating laccase production in *T. versicolor*.

#### 3.1.3 Effect of initial medium pH on laccase production

The effect of initial medium pH on laccase production is shown in Figure 1C. Laccase activity increased as the pH rose from 2.5 to 5.5 and decreased at higher pH values, with the highest activity observed at pH 5.5. This pattern indicates that the optimal pH for laccase production is around 5.5, which is consistent with the typical pH range for fungal growth and enzyme secretion (33). At both lower and higher pH values, laccase activity decreased, likely due to adverse effects on fungal growth and enzyme stability (38). Similar observations have been reported in other studies, highlighting medium pH as a critical factor influencing fungal laccase production (39, 40). Therefore, optimizing medium pH is essential to maximize laccase production.

#### 3.1.4 Effect of medium volume on laccase production

The effect of medium volume on laccase production is presented in Figure 1D. Laccase activity increased as the medium volume rose from 40 to 100 mL and declined at higher volumes, reaching a maximum at 100 mL. This trend reflects a balance between oxygen transfer efficiency and physical limitations on mycelial growth in submerged fermentation (41). At low volumes, despite relatively high oxygen availability, limited growth space and a shallow liquid layer may increase mycelial susceptibility to mechanical shear, thereby impairing enzyme secretion (42). In contrast, excessive medium volumes increase liquid depth and reduce oxygen transfer efficiency, thereby restricting the growth of this aerobic fungus and consequently decreasing laccase production (43, 44). Similar trends have been observed in other fungal systems, suggesting that medium volume plays a critical role in enzyme secretion (45, 46), possibly through its influence on oxygen transfer. Therefore, optimizing medium volume is essential for maximizing laccase production.

### 3.2 Box–Behnken optimization and model validation of laccase production

#### 3.2.1 Regression equation for laccase production

The experimental results of the Box–Behnken design are presented in Table 2. Based on these data, a second-order polynomial model was developed using Design-Expert software to describe the relationship between the independent variables (fermentation time, initial medium pH, and medium volume) and laccase activity. The fitted regression (Equation 2) in terms of coded variables is as follows:


Y(laccaseactivity,U/L)=6737.58-162.05X-1171.89X+2 (2)

902.85X+328.94XX1-296.07XX1+341.67XX2-3

1216.42X-12883.06X-223051.05X23

The ANOVA results are summarized in Table 3. The model was statistically significant (*p* < 0.05), while the lack-of-fit was not significant (*p* = 0.2136), indicating good model adequacy within the studied range. The high coefficient of determination (*R*^2^ = 0.9960) and adjusted coefficient of determination (adjusted *R*^2^ = 0.9909) indicate good agreement between predicted and experimental values. In addition, the low coefficient of variation (CV = 4.26%) suggests good reproducibility. Overall, the model is reliable for predicting laccase activity in *T. versicolor* strain Tv-1.

**TABLE 3:** Analysis of variance for the quadratic regression model.

Variance source	Sum of squares	Degree of freedom	Mean square	*F*-value	*P*-value	Comment
Model	5.95 × 10^7^	9	6.61 × 10^6^	195.56	< 0.0001	*
*X*_1_	2.10 × 10^5^	1	2.10 × 10^5^	6.21	0.0415	*
*X*_2_	2.36 × 10^5^	1	2.36 × 10^5^	6.99	0.0333	*
*X*_3_	6.52 × 10^6^	1	6.52 × 10^6^	192.80	< 0.0001	*
*X*_1_ *X*_2_	3349.52	1	3349.52	0.10	0.7622	NS
*X*_1_ *X*_3_	36919.70	1	36919.70	1.09	0.3309	NS
*X*_2_ *X*_3_	6945.56	1	6945.56	0.21	0.6641	NS
*X*_1_^2^	6.23 × 10^6^	1	6.23 × 10^6^	184.20	< 0.0001	*
*X*_2_^2^	3.28 × 10^6^	1	3.28 × 10^6^	97.07	< 0.0001	*
*X*_3_^2^	3.92 × 10^7^	1	3.92 × 10^7^	1158.86	< 0.0001	*
Residual	2.37 × 10^5^	7	33822.60			
Lack of fit	1.51 × 10^5^	3	50359.74	2.35	0.2136	NS
Pure error	85679.00	4	21419.75			
Corrected total sum of squares	5.98 × 10^7^	16				

*Statistically significant (*p* < 0.05). NS, Not significant (*p* > 0.05).

#### 3.2.2 Effect of independent variables on laccase production

As shown in Table 3, the linear terms *X*_1_, *X*_2_, and *X*_3_, as well as their quadratic terms (*X*_1_^2^, *X*_2_^2^, and *X*_3_^2^), significantly affected laccase production, while the interaction terms (*X*_1_*X*_2_, *X*_1_*X*_3_, and *X*_2_*X*_3_) were not significant. Based on the *F*-values, medium volume had the greatest effect, followed by initial medium pH, whereas fermentation time showed the least effect.

**FIGURE 2:**
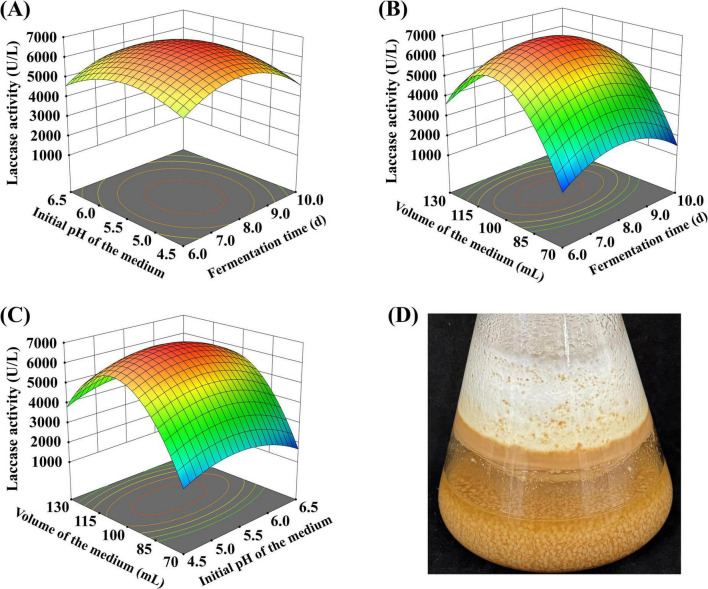
Response surface plots showing interactions among independent variables affecting laccase production by *T. versicolor* strain Tv-1 **(A–C)** and the fermentation broth obtained from the validation experiment **(D)**.

Three-dimensional response surface plots (Figure 2) were used to illustrate the combined effects of the variables, with one factor fixed at its center level. The surfaces exhibited pronounced curvature, indicating a quadratic relationship between the variables and laccase activity, with a distinct maximum within the experimental range.

Among the variable combinations, medium volume showed a greater effect in combination with fermentation time (Figure 2B) and initial medium pH (Figure 2C), whereas the interaction between fermentation time and pH was comparatively weak (Figure 2A). These observations are consistent with the ANOVA results, further conforming the adequacy of the regression model.

#### 3.2.3 Model validation and confirmation experiments

The regression model predicted the optimal conditions for laccase production by *T. versicolor* strain Tv-1 as a fermentation time of 7.9 days, an initial medium pH of 5.41, and a medium volume of 104.47 mL. For practical feasibility, these values were adjusted to 7.9 days, pH 5.4, and 105 mL. Under these conditions, the predicted laccase activity was 6818.73 U/L. Validation experiments (Figure 2D) performed in triplicate yielded an average laccase activity of 6843.14 ± 34.96 U/L, which is in good agreement with the predicted value. These results confirm the reliability and predictive accuracy of the regression model.

### 3.3 Purification of *T. versicolor* laccase and electrophoretic analysis

#### 3.3.1 Ammonium sulfate fractional precipitation of *T. versicolor* laccase

**FIGURE 3:**
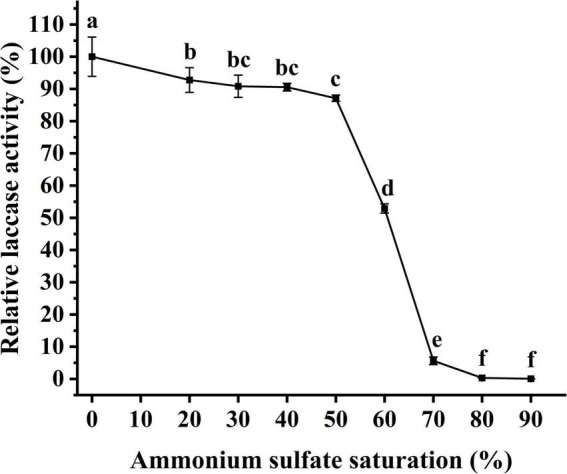
Ammonium sulfate fractional precipitation of laccase from *T. versicolor*. Different letters indicate significant differences (*p* < 0.05, Duncan’s multiple range test).

The effect of ammonium sulfate saturation on laccase precipitation is shown in Figure 3. As saturation increased from 0 to 50%, the relative laccase activity in the supernatant decreased gradually but remained high, indicating that only a small amount of laccase was precipitated in this range. In contrast, a marked decrease ( > 80%) was observed between 50 and 70% saturation, suggesting that most laccase precipitated within this range, whereas proteins precipitated below 50% were primarily non-laccase components. When the saturation reached 70%, the residual activity in the supernatant was below 6%, indicating that the majority of laccase had precipitated. Accordingly, 50 and 70% saturation levels were selected as the first- and second-stage salting-out levels, respectively. This two-step strategy effectively removed non-laccase proteins in the initial stage and enriched laccase in the subsequent fraction. The resulting precipitate was dissolved in PB to obtain the laccase preparation S2.

#### 3.3.2 Anion-exchange purification of dialyzed laccase

**FIGURE 4:**
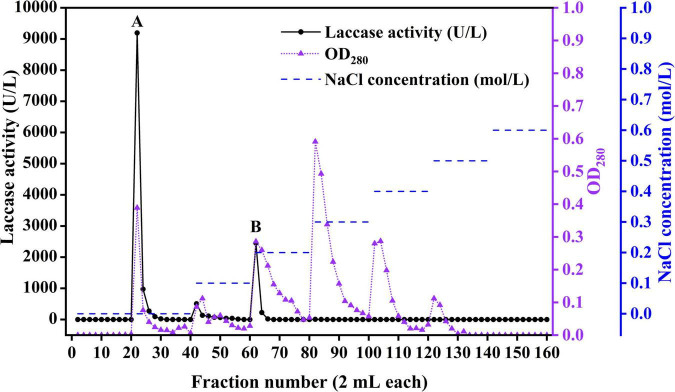
Elution profile of *T. versicolor* laccase on a DEAE-Sepharose 6 FF anion-exchange column. Laccase activity (U/L), OD_280_, and NaCl concentration (mol/L; stepwise gradient) are shown.

The dialyzed laccase preparation (S3) was subjected to anion-exchange chromatography, with the elution profile shown in Figure 4. Two distinct activity peaks were detected, corresponding to two laccase isoforms from *T. versicolor*. The first peak (LacA) was eluted without NaCl, indicating a lower affinity for the anion-exchange resin, while the second peak (LacB) was eluted at 0.2 mol/L NaCl, reflecting stronger binding to the resin. This separation is attributed to differences in net charge and binding affinity toward the DEAE-Sepharose matrix (47). The distinct elution behaviors of LacA and LacB further support structural or electrostatic differences between the isoforms, consistent with previous reports on multiple laccase isoforms resolved by anion-exchange chromatography (48, 49).

#### 3.3.3 SDS-PAGE and native-PAGE analyses of laccase samples

**FIGURE 5:**
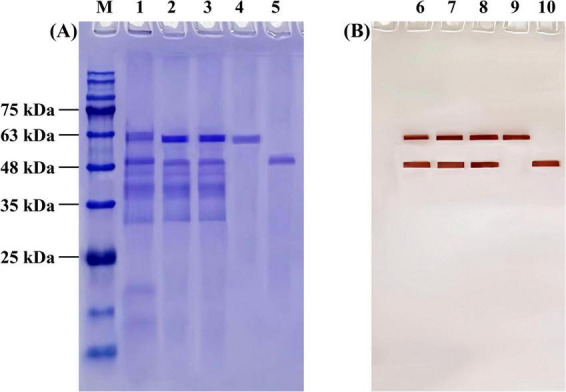
SDS-PAGE **(A)** and native-PAGE **(B)** profiles of *T. versicolor* laccase. Lanes 1–5: S1, S2, S3, LacA, and LacB, respectively; lanes 6–10: S1, S2, S3, LacA, and LacB, respectively.

The SDS-PAGE and native-PAGE profiles of *T. versicolor* laccase preparations (S1, S2, S3, LacA, and LacB) are shown in Figure 5. SDS-PAGE analysis revealed that the crude preparation (S1) exhibited multiple protein bands, indicating a complex protein composition. After ammonium sulfate precipitation and dialysis, the number of bands in S2 and S3 decreased, suggesting the removal of non-laccase proteins and the enrichment of laccase. Following anion-exchange chromatography, both LacA and LacB displayed single protein bands, confirming the successful purification of two distinct laccase isoforms with different molecular weights (Figure 5A).

Native-PAGE further confirmed the presence of two laccase isoforms, with LacA and LacB each exhibiting a single distinct band (Figure 5B). These results indicate structural or electrostatic differences between the isoforms, consistent with previous reports on multiple laccase isoforms in *T. versicolor* (50).

The molecular weights of LacA and LacB were estimated to be 62.87 and 54.12 kDa, respectively. LacA falls within the typical range reported for *T. versicolor* laccases (60–66 kDa) (33, 51), whereas LacB was slightly smaller than this range. Higher molecular weights (97–100 kDa) have also been reported (52, 53). Such variation may be attributed to differences in strain, culture medium (54), or the degree of glycosylation (34), which can influence the molecular size of laccases.

#### 3.3.4 Purification summary of *T. versicolor* laccase

The purification results are summarized in Table 4. Throughout the purification process, total protein, total laccase activity, and recovery decreased, while specific activity and purification fold increased, indicating progressive enrichment of laccase. After ammonium sulfate precipitation and dialysis, laccase loss was relatively low, with most enzymatic activity retained. In contrast, a greater loss of laccase activity occurred during the anion-exchange chromatography step. Nevertheless, this step successfully separated two distinct laccase isoforms, LacA and LacB.

**TABLE 4:** Purification of laccase from *T. versicolor* strain Tv-1.

Purification steps		Total protein (mg)	Total activity (U)	Specific activity (U/mg protein)	Purification (fold)	Recovery (%)
Crude laccase preparation (S1)		97.08	641.72	6.61	1.00	100%
Ammonium sulfate precipitation (S2)		69.64	502.82	7.22	1.09	78%
Dialysis (S3)		42.31	444.48	10.51	1.59	69%
DEAE-Sepharose	LacA	1.18	39.17	33.07	5.00	6%
	LacB	0.89	12.01	13.47	2.04	2%

Given their high purity, LacA and LacB are suitable for enzymatic characterization. The crude laccase preparation (S1), which is more readily available and cost-effective, may be more suitable for practical applications. Therefore, the optimum temperature, thermal stability, optimum pH, and pH stability of S1, LacA, and LacB were evaluated.

### 3.4 Optimum temperature and pH, and stability of *T. versicolor* laccase

#### 3.4.1 Optimum temperature and thermal stability

**FIGURE 6:**
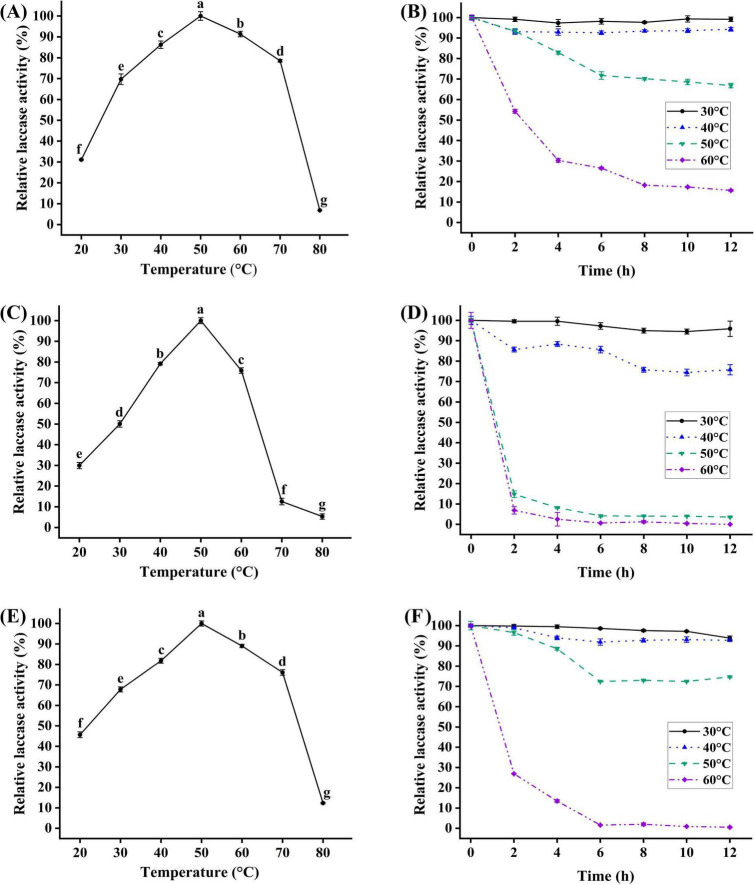
Optimum temperature and thermal stability of *T. versicolor* laccase S1 (A,B), LacA (C,D), and LacB (E,F). Different letters indicate significant differences (*p* < 0.05, Duncan’s multiple range test).

The optimum temperature and thermal stability of laccase samples S1, LacA, and LacB are presented in Figure 6. The relative activity of all three samples exhibited a typical bell-shaped response to increasing temperature. As the temperature increased from 20 to 50°C, relative laccase activity increased, reaching a maximum at 50°C, consistent with previous reports on *T. versicolor* laccases (55). However, further increases from 50 to 80°C led to a decline in activity, likely due to thermal denaturation resulting in loss of enzymatic structural integrity (56).

Temperature also significantly affected the thermal stability of *T. versicolor* laccase (Figure 6). Over a 12-h period, all three samples maintained good stability at 30°C. At 40°C, samples S1 and LacB demonstrated good heat tolerance, with < 8% loss in relative activity. In contrast, LacA exhibited a more pronounced activity loss, exceeding 24% under the same conditions, indicating its lower thermal stability at elevated temperatures. This differential stability became more evident at higher temperatures (50 and 60°C), where LacA consistently displayed the greatest activity loss. These results suggest that LacB possesses better thermal stability than LacA, likely due to intrinsic differences between the isoforms. Overall, the relative activity of laccase decreased with increasing temperature, confirming its temperature-sensitive nature, consistent with previous studies (34).

Although the optimum temperature for *T. versicolor* laccase was found to be 50°C, its thermal stability at this temperature was significantly reduced, particularly for the LacA isoform. Since all three samples maintained high thermal stability at 30°C while retaining good catalytic activity, 30°C was selected for subsequent mycotoxin degradation experiments. This strategy prioritizes sustained enzymatic stability over maximum initial reaction rate, which is crucial for efficient degradation of multiple mycotoxins during prolonged incubation.

#### 3.4.2 Optimum pH and pH stability

**FIGURE 7:**
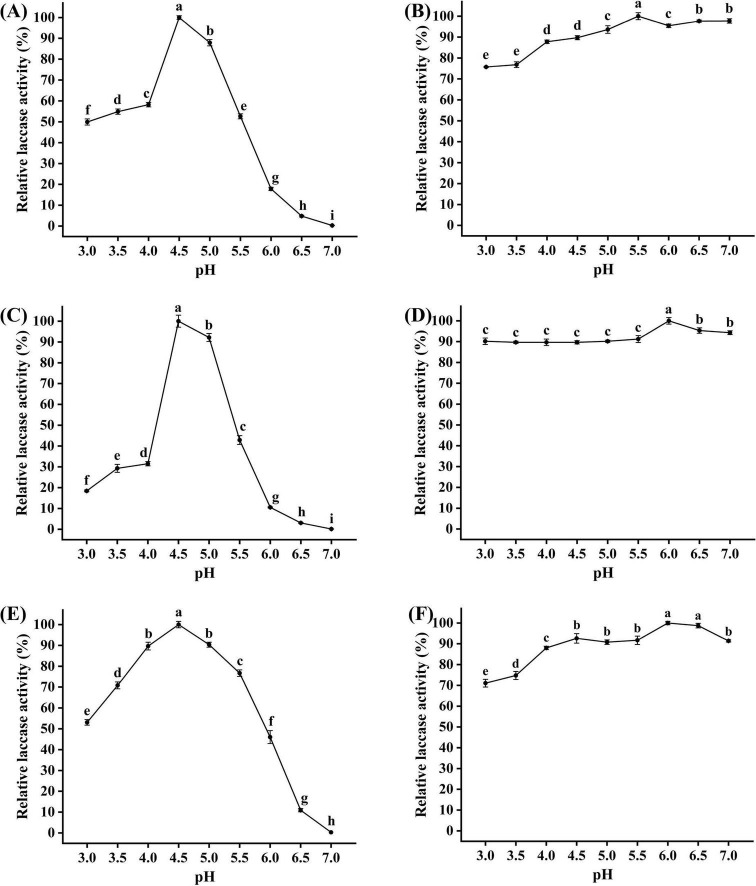
Optimum pH and pH stability of *T. versicolor* laccase S1 (A,B), LacA (C,D), and LacB (E,F). Different letters indicate significant differences (*p* < 0.05, Duncan’s multiple range test).

The effects of pH on the relative laccase activity of samples (S1, LacA, and LacB), as well as their pH stability, are shown in Figure 7. All three samples exhibited maximum activity at pH 4.5, consistent with the general characteristic that fungal laccases typically display optimal activity under acidic conditions (55, 57). As the pH deviated from the optimum, relative laccase activity decreased significantly. This reduction can be attributed to the adverse effects of pH on the enzyme’s structural conformation and the ionization state of the substrate, both of which affect enzyme–substrate interactions and catalytic efficiency (58, 59). Previous studies have reported optimum pH values of 4.0 or 5.0 for *T. versicolor* laccases (34, 60), which are close to the optimum pH of 4.5 observed in this study. The minor discrepancies are likely due to differences in fungal strains and variations in the substrates used for laccase activity assays, such as guaiacol, ABTS, or syringaldazine (51, 60, 61).

The pH stability profiles further highlight the strong acid tolerance of the three samples (Figure 7). After 24 h of incubation across a pH range of 3.0–7.0, the relative activities of S1, LacA, and LacB remained above 75, 89, and 71%, respectively, indicating that LacA exhibited greater acid tolerance than LacB under the tested conditions. Notably, after incubation at the optimum pH of 4.5 for 24 h, all three samples retained more than 89% of their initial activity, demonstrating that *T. versicolor* laccase possesses excellent acid resistance at its optimum pH. Given both the optimum pH and pH stability of *T. versicolor* laccase, a reaction system with a pH of 4.5 is recommended for mycotoxin degradation.

### 3.5 Degradation of AFB_1_, ZEN, and DON by *T. versicolor* laccase

**FIGURE 8:**
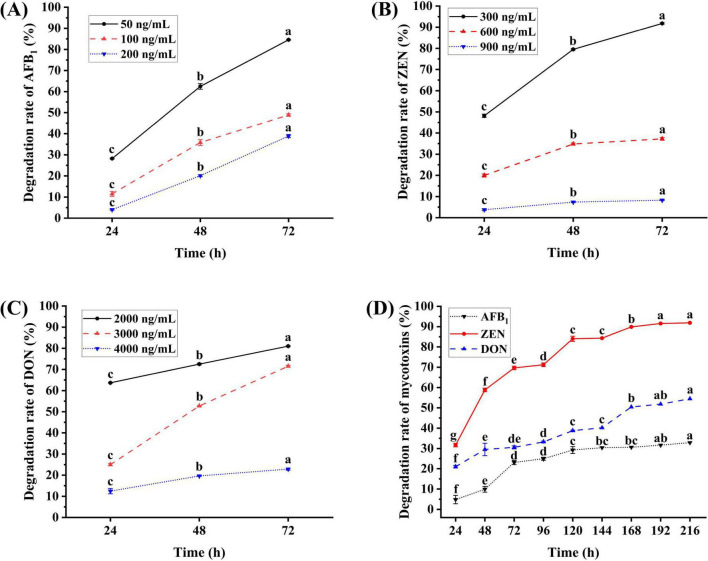
Individual degradation of AFB_1_ (A), ZEN (B), and DON (C) by *T. versicolor* laccase, and simultaneous degradation of the three mycotoxins (D). Different letters on the same line indicate significant differences (*p* < 0.05, Duncan’s multiple range test).

The degradation of individual mycotoxins is shown in Figures 8A–C. Laccase degraded AFB_1_, ZEN, and DON under single-mycotoxin conditions, with degradation rates increasing over time, consistent with previous studies (12, 62). Degradation efficiency was concentration-dependent, with higher initial concentrations of mycotoxins resulting in lower degradation rates. This could be due to enzyme saturation and inhibitory effects of the mycotoxins and their transformation products. Additionally, organic solvents in the reaction system may reduce degradation rates by weakening laccase activity (63). In the AFB_1_ and DON degradation systems, laccase activity was less affected by methanol and acetonitrile, because their concentrations did not exceed 5%. However, in the ZEN degradation system, where acetonitrile concentrations ranged from 7.5 to 22.5%, laccase activity was significantly impacted, leading to a notable reduction in ZEN degradation rates. These findings further support the notion that organic solvents, particularly at higher concentrations, can interfere with enzyme activity (64, 65), thereby reducing mycotoxin degradation efficiency. An inverse relationship between the initial concentration of AFB_1_ and its degradation rate has also been reported (66). Moreover, when substrate concentrations are excessively high, the reaction system may approach saturation, causing the enzymatic reaction rate to level off rather than increase proportionally (62). Therefore, in practical applications, efficient detoxification may require adjusting enzyme dosage, extending treatment duration, and minimizing the interference of organic solvents based on the initial contamination level.

Given that *T. versicolor* laccase was able to degrade each of the three mycotoxins individually, its capability for simultaneous degradation of AFB_1_, ZEN, and DON was further investigated. As shown in Figure 8D, *T. versicolor* laccase successfully degraded all three mycotoxins simultaneously. During the incubation period from 24 to 192 h, the degradation rates of AFB_1_, ZEN, and DON increased significantly with time. However, after 192 h, the degradation rates of all three mycotoxins showed no further significant improvement, suggesting that the reaction gradually reached a plateau. At 216 h, the degradation rates of AFB_1_, ZEN, and DON reached 32.88, 91.88, and 54.45%, respectively. The sustained degradation over the extended incubation period may be attributed to the good stability and catalytic activity of the laccase under the selected reaction conditions (30°C and pH 4.5). Notably, when all three mycotoxins coexisted in the reaction system, the degradation efficiency of each mycotoxin was lower than in the corresponding single-mycotoxin system, suggesting that the coexistence of multiple mycotoxins may interfere with the enzymatic degradation process. Similar phenomena have been reported in studies involving the simultaneous degradation of six different mycotoxins (67), possibly due to competitive interactions or mutual interference among substrates during enzymatic catalysis. However, the underlying mechanisms responsible for this effect remain to be fully elucidated.

In both the individual mycotoxin degradation experiments and the simultaneous degradation experiments, heat-inactivated S3 did not significantly degrade any of the mycotoxins. For example, after 72 h of treatment with heat-inactivated S3, the residual concentrations of AFB_1_ were 49.37 ± 0.48, 98.05 ± 1.45, and 198.38 ± 1.76 ng/mL at initial concentrations of 50, 100, and 200 ng/mL, respectively. The residual concentrations of ZEN were 290.99 ± 1.03, 587.65 ± 5.62, and 869.32 ± 6.78 ng/mL at initial concentrations of 300, 600, and 900 ng/mL, respectively. The residual concentrations of DON were 1969.98 ± 22.21, 2948.98 ± 35.59, and 3955.37 ± 39.31 ng/mL at initial concentrations of 2,000, 3,000, and 4,000 ng/mL, respectively. These results indicate that the observed mycotoxin degradation was mainly associated with active laccase.

Although the present results demonstrate that S3 was able to degrade AFB_1_, ZEN, and DON both individually and simultaneously, isoform-specific degradation activity was not investigated in this study. Because LacA and LacB were obtained with low recovery yields after anion-exchange purification, the degradation assays were performed using S3 rather than the individual purified isoforms. Therefore, the relative contributions of LacA and LacB to AFB_1_, ZEN, and DON degradation remain unknown. This is an important limitation of the present study and should be addressed in future work using sufficient amounts of purified LacA and LacB.

In practical agricultural production, crops are often contaminated by multiple mycotoxins. Laccase, due to its broad substrate spectrum, has gained attention for multi-mycotoxin degradation. Previous studies have shown that laccase can degrade AFB_1_ by disrupting its furo-furan ring and lactone ring, thereby reducing its toxicity (68). For ZEN, laccase catalyzes hydroxylation at the C15 position, forming 15-OH-ZEN, a less toxic derivative (67). Furthermore, degradation products of AFB_1_ and ZEN show no significant toxicity to mouse macrophages (69), supporting enzymatic detoxification. While DON is more difficult to degrade (19), *T. versicolor* laccase can degrade 43–61% of DON in the presence of a chemical mediator (21). Laccase from *Weizmannia coagulans* (Lac-W) was reported to degrade DON directly without a mediator, although its efficiency was low and could be improved with a mediator (67, 70). At the mechanistic level, *T. versicolor* laccase oxidizes alcohol groups at the C3 and C15 positions of DON, converting them into ketones and forming 3,15-diketodeoxynivalenol (21). In this study, the laccase from *T. versicolor* strain Tv-1 successfully degraded AFB_1_, ZEN, and DON directly and simultaneously without mediators at 30°C, offering a promising and cost-effective solution for detoxifying multiple mycotoxins in contaminated agricultural commodities.

## 4 Conclusion

The fermentation conditions for laccase production by *Trametes versicolor* strain Tv-1 were systematically optimized through single-factor experiments and response surface methodology. Following downstream separation and purification, SDS-PAGE and native-PAGE analyses confirmed that *T. versicolor* strain Tv-1 secreted two laccase isoforms, designated LacA and LacB. Enzymatic characterization determined the optimum temperature and pH, as well as the thermal and pH stability of the laccase preparation. Considering both enzyme stability and catalytic performance, 30°C and pH 4.5 were identified as suitable conditions for mycotoxin degradation. Under these conditions, the laccase degraded the individual mycotoxins (AFB_1_, ZEN, and DON) and also simultaneously degraded all three in a mixed system.

Notably, higher initial mycotoxin concentrations and the coexistence of multi-mycotoxins reduced degradation efficiency, highlighting potential limitations that need to be addressed for practical applications. Overall, the naturally occurring *T. versicolor* laccase provides a sustainable, low-temperature, and mediator-free strategy for the simultaneous degradation of multiple mycotoxins. Future work should focus on elucidating the mechanisms underlying reduced degradation efficiency at high mycotoxin loads and during multi-mycotoxin coexistence, determining the isoform-specific contributions of LacA and LacB to mycotoxin degradation, identifying transformation products and evaluating their residual toxicity, and validating performance in real food/feed matrices under scalable processing conditions. In addition, cloning and heterologous expression of *T. versicolor* laccase genes, together with enzyme engineering to improve catalytic efficiency and robustness, will be key steps toward cost-effective industrial application.

## Data Availability

The original contributions presented in the study are included in the article/supplementary material, further inquiries can be directed to the corresponding author.
